# Outstanding micro-endemism in New Caledonia: More than one out of ten animal species have a very restricted distribution range

**DOI:** 10.1371/journal.pone.0181437

**Published:** 2017-07-20

**Authors:** Maram Caesar, Philippe Grandcolas, Roseli Pellens

**Affiliations:** Institut de Systématique, Evolution, Biodiversité –Muséum national d’Histoire naturelle, CNRS UPMC EPHE, Sorbonne Universités, CP 50, 45, rue Buffon, Paris, France; University of Innsbruck, AUSTRIA

## Abstract

New Caledonia is a biodiversity hotspot, with an extremely high number of endemic species with narrow distribution ranges that are at high risk of extinction due to open-cast nickel mining, invasive species and seasonal man-induced fires. Mentions of micro-endemism permeate the literature on the biota of this archipelago. However, so far there has been no research comparing distribution range in different animal groups. The aim of this study is to examine the implication of different sampling effort variables in order to distinguish micro-endemicity from data deficiency, and evaluate the distribution range, frequency, and extent to which micro-endemism is common to several groups of organisms. We compiled a dataset derived from publications in *Zoologia Neocaledonica*, comprising 1,149 species, of which 86% are endemic to New Caledonia. We found that the sampling effort variables that were best correlated with distribution range were the number of sampling dates and the number of collectors *per* species. The median value of sampling dates was used to establish a cut-off point for defining adequately sampled species. We showed that, although only 52% of species were sampled adequately enough to determine their distribution range, the number of species with a very narrow distribution range was still high. Among endemics from New Caledonia, 12% (116 species) have ranges ≤5.2km^2^ and 3.9% (38 species) have ranges between 23 and 100 km^2^. Surprisingly, a similar trend was observed in non-endemic species: 22% occurred in areas ≤ 5.2 km^2^, and 8% in areas 23–100 km^2^, suggesting that environmental dissimilarity may play an important role in the distribution of these species. Micro-endemic species were predominant in 18 out of 20 orders. These results will contribute to a re-assessment of the IUCN red list of species in this archipelago, indicating that at least 116 species are probably critically endangered.

## Introduction

Micro-endemism, short-range endemism, narrow endemism, restricted distribution range are terms commonly and interchangeably used to describe very small geographical distribution ranges. This is a common form of rarity [1, 2], and the literature has plenty of examples of micro-endemic species and of sites that harbor exceptional rates of micro-endemism [3–5]. Despite its commonness, the quantification of micro-endemism is quite challenging, particularly because the very characterization of a distribution range can be obscured by scaling effects [6] and by differential sampling efforts in space and time, which might lead to a confusion between micro-endemicity and data deficiency.

The massive destruction and transformation of natural habitats [7] is leading to a major crisis in the history of life on Earth [8]. Therefore, characterizing species that are micro-endemic and sites that harbor important rates of micro-endemism is fundamental for establishing adequate conservation strategies for reducing major biodiversity losses and protecting several important and vulnerable functions in the ecosystem [9]. From a conservation perspective, the principal particularity of micro-endemicity is that the population size is often very small (even when density is locally high). As a consequence, a micro-endemic species’ survival is highly dependent on the long-term permanence and integrity of very localized habitats.

Although micro-endemic species can be found virtually anywhere, they are particularly abundant in some regions known for their rich and original biota derived from local evolution and diversification (e.g., the Cape flora, the mammals and frogs of Madagascar) [3–5, 10], or in some environments characterized by strong discontinuity such as, for example, limestone karsts [11,12]. These local diversification bursts have led to biotas that are rich in species with very restricted geographical ranges or with marked differences in the way they exploit local resources.

Among these remarkable regions of the world, New Caledonia is not only famous for its exceptional regional endemism [13] but also for its extremely high local micro-endemism [13–16]. New Caledonia is the oldest oceanic island [17,18]. It is considered a biodiversity hotspot due to its magnificent flora and fauna, which is characterized by a high number of locally endemic species that are under threat due to nickel mining, repeated fires, and invasive species [10, 19–23]. Mentions of micro-endemicity permeate the literature on the New Caledonian fauna and flora. For instance, studies of local insect diversification reveal that most endemic species are allopatric or secondarily sympatric on different small mountains [24–27], or in single creeks [28]. A new harvestman species was found at each newly explored locality [29]. Forty eight percent of land snails studied [30] were found in a single locality; and gecko species from the genus *Dierogekko* were each found to be restricted to a single site, sometimes very close to one harboring a different species, or isolated without any visible geographical barrier [31]. The same was reported for the flora, with twenty one percent of species defined as micro-endemic [32].

Repeated documentation of micro-endemism in several components of the New Caledonia biota highlights a clear need for carrying out a synthesis aimed at understanding not only its frequency, distribution area and main drivers, but also the extent to which it actually occurs in different groups of organisms. Such a study was carried out by Wulff *et al*. [32], providing data for understanding micro-endemicity within the New Caledonian flora. However, despite the diversity and richness of the local fauna, which has been documented in many taxonomic studies e.g. [16, 33–34], nothing has yet been done to advance our knowledge of the occurrence of micro-endemism in animals. In order to contribute to this task we designed a study for assessing the extent to which micro-endemicity is common within the New Caledonian fauna, and distinguish micro-endemism from sampling deficiency. More specifically, we defined different distribution range classes for New Caledonia and explored (1) the frequency of species in these classes, (2) a set of sampling effort variables in order to find the ones that were more likely to have implications in determining a species distribution range; and (3) we investigated whether micro-endemism significantly occurred in a set of well-sampled species from diverse taxonomic groups. In addition to that, we also searched for the status of these species in IUCN red list. As our dataset also comprised non-endemic species, we compared them to endemics in order to determine the extent to which these distributional patterns might result from local evolution.

## Material and methods

### Study site

New Caledonia is an oceanic archipelago in the Pacific Ocean located between 18°–23° S and 163°–169° E. It is approximately 1,500 km away from Australia, the nearest continent. It comprises a main island (Grande Terre), considered to be the oldest oceanic island (37Ma) [17–18], and several smaller and much more recent islands, making a total land surface of about 18,760 km^2^. Grande Terre is a long island oriented northwest-to-southeast with a surface area of 16,372 km^2^ (350 km in length and 50–70 km in width). Its landscape is characterized by a mountain range running along the length of the island, with five peaks over 1,500 m and a complex series of more or less connected very steeply sloped small mountains.

### The dataset

We based this study on species studied in *Zoologia Neocaledonica*, an ongoing series of 8 volumes on the taxonomy of the New Caledonian fauna, published by the Muséum National d’Histoire naturelle in Paris. We used data published between 1988 and 2014 [33–40], the period with the highest number of publications on the systematics of New Caledonia [27]. *Zoologia Neocaledonica* is dedicated to the description of species, the revision of genera, and to reporting the natural history of terrestrial and freshwater animals occurring in New Caledonia. The volumes comprise 105 chapters, and represent 25% of publications on this subject over this period. In terms of number of publications, *Zoologia Neocaledonica* is followed by Zootaxa with 12% (50 publications), and Zoosystema with 6% (26 publications). The remaining publications over the same period of time are spread out between 117 other journals.

We built a database with 9,818 entries, in which we recorded all available information on the taxonomy (order, family, genus, species and any change in name or classification), occurrence (geographical coordinates, locality, altitude, type of ecosystem, soil, and other environmental characteristics) and collection (sampling dates, name of each collector) of each specimen. This dataset was complemented by an exhaustive search using the species name as query (using all names and combinations when there were taxonomic changes) in Web of Science, Zoological Records and Google Scholar using the following Query terms: Topic: ‘new species’ AND Topic: ‘new caledonia’ OR Topic: (new genus) AND Publication Name: (new caledonia) NOT Topic: ‘plant*’ NOT Title: ‘bryophyte’ NOT Topic: ‘moss’ NOT Title: ‘fungi’ NOT Topic: ‘bacteria’ NOT Publication Name: ‘Memoires du Museum National d'Histoire Naturelle’ NOT Topic: ‘tree*’ NOT Title: ‘annonaceae’ NOT Title: ‘lagoons’ NOT Title: deep water NOT Title: marine NOT Publication Name: ‘Systematic Botany’ NOT Publication Name: ‘New bryophyte’ Timespan: 1988–2014. This was followed by a search in Google Scholar and Google using the name of the species and New Caledonia.

When information on sampling efforts was absent, we assumed the existence of one collector and one collection date for each sample. For specimens collected with Malaise traps or other long-stay capture methods, we assigned a single sampling date independently of the number of days the trap was active.

#### Estimating distribution range, sampling effort and accessing conservation status

The distribution range of each species was calculated using the convex hull method in ArcGis 10.2. The convex hull calculates the minimum convex polygon that encompasses all available records for a given species. It was used here for all species with at least three records at different points. For species with only one point of occurrence we assigned a value of 1/3 of the smallest area estimated with the Convex Hull. For those with two points, the area was calculated as follows:

$$\frac{mA}{(\frac{1}{D})*mA}$$

where *mA* is the minimum area estimated with a polygon, and D is the distance between the two points of occurrence.

For 130 species that had at least 15 distinct records of occurrence, we also estimated the area with MaxEnt 3.3.3 [41], using a polygon of the New Caledonia archipelago as a mask. Environmental data were obtained from the WORLDCLIM Version 1.4 database (http://www.worldclim.org; [42]), at the highest resolution (30arc-seconds (~1 km)). Analyses were carried out with 19 Bioclim variables plus altitude. Comparison with the convex hull showed that the areas estimated with MaxEnt were much smaller (t = 16.5, df = 131.5, p-value < 2.2e-16). For this reason, and considering that the minimum convex polygon method is the one used by the IUCN for defining the Area of Occupancy (AOO) and the Extent of Occurrence (EOO) [43], we based our analysis on results obtained by the convex hull method.

Six sampling effort variables were examined in order to assess their implications on distribution range estimates. The first three described the sampling effort actually employed for sampling each species: the number of sampling dates, the number of collectors that sampled the species, and the number of specimens *per* species. The other three represented inferences on the possibility of being collected: the number of times a species’ collector was in the field, the number of points sampled by a species’ collector, and the mean area of the polygons sampled by a species’ collector.

Threat status was accessed from the IUCN red list at http://www.iucnredlist.org in December 27^th^ 2016 [44].

## Results

### a) Characterization of the dataset

Our dataset comprised 1,149 species belonging to 352 genera, 91 families, and 20 orders (11 insect orders and 9 orders from very distantly related organisms such as Squamata, Araneae, and Mollusca). Forty four percent of genera and 59% of species were newly described. 78% of all species and 90% of newly described species were only known from *Zoologia Neocaledonica* without any other mention in the literature. Almost all new species (98%) were known only from New Caledonia. Sampling involved 358 collectors, 41,289 specimens, and 1,803 different sampling points (Table 1). Species distribution area ranged from a single point to 11,500 km^2^.

**Table 1:** Characterization of the dataset.

Number of genera	352	
Number of new genera	154	44%
Total number of species	1149	
Number of new species	674	59%
Number of species cited only in *Zoologia Neocaledonica*	893	78% ^a^
Number of species also cited in another publication	257	22% ^a^
Number of new species cited only in *Zoologia Neocaledonica*	604	90% ^b^
Number of species endemic to New Caledonia	982	86% ^a^
Number of species also found elsewhere–Non Endemic	167	14% ^a^
Number of new species endemic to New Caledonia	660	98% ^b^
Number of specimens	41289	
Number of collection points	1803	
Number of collectors	358	

^a^ percentage of the total number of species

^b^ percentage of new species.

**Fig 1:**
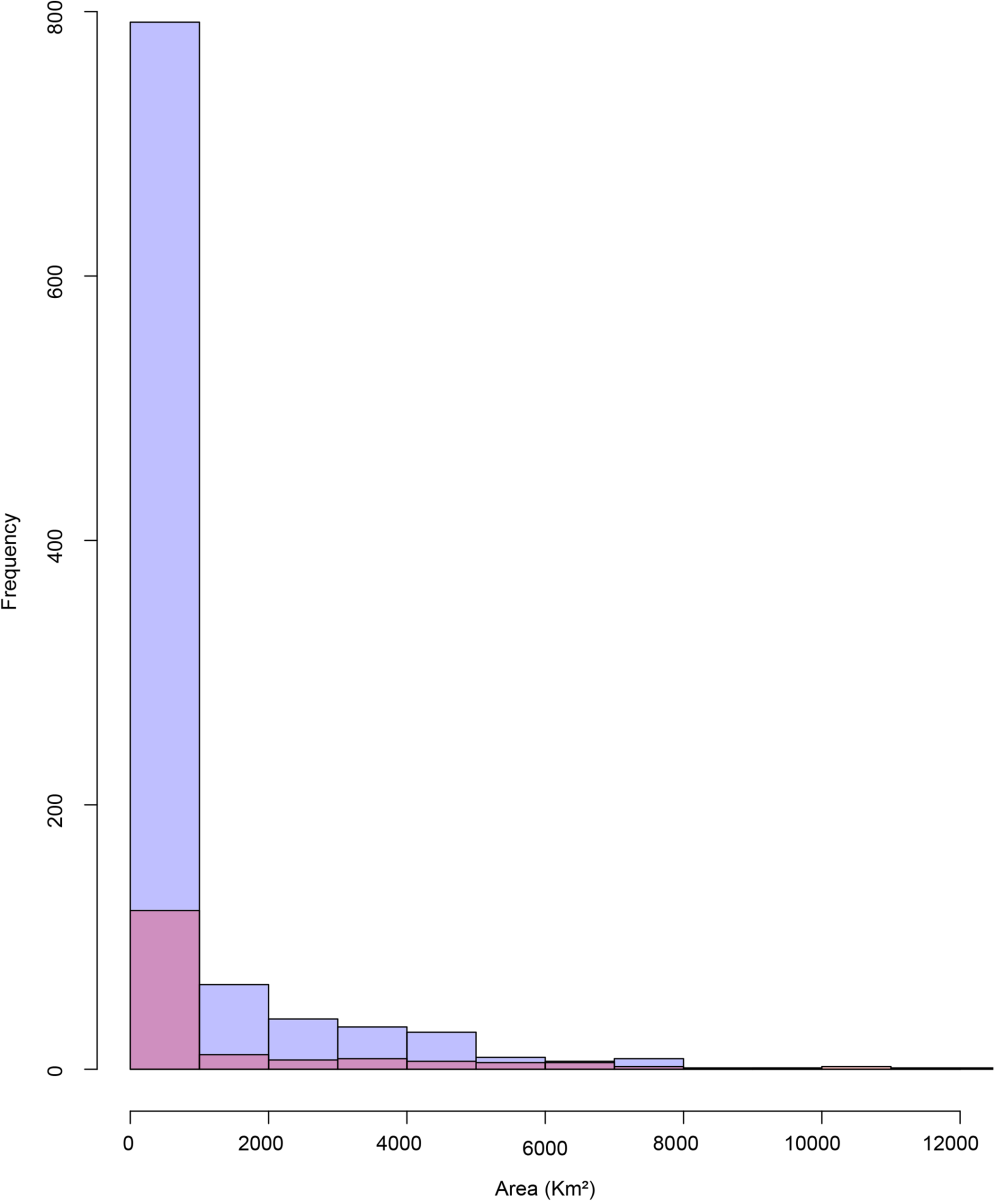
Frequency of endemic (violet) and non-endemic (light pink) species in different classes of distribution range.

Despite differences in total numbers of endemic and non-endemic, species with narrow distribution ranges were the most frequent in the dataset (Fig 1).

### b) Main trends in species that are endemic and non-endemic to New Caledonia

982 (86%) species were known only from the New Caledonian archipelago, and therefore considered regionally endemic, whereas 167 (14%) species were also recorded elsewhere. On average, the distribution area was significantly lower for species endemic to New Caledonia (less than half that of non-endemics), although the sampling effort was similar in the two sets (Table 2). For both sets of species, the median was consistently much lower than the mean, indicating that distribution area and sampling effort are highly skewed to the lowest values.

**Table 2:** Area of distribution and values of different sampling effort variables for species endemic and non-endemic to New Caledonia. The comparison was made with a Student t test and significant values are indicated in bold.

	Endemic		Non-Endemic			All species	
	Mean ±SE	Median	Mean ±SE	Median	t Test	Mean ±SE	Median
Area (km^2^)	693 ±46	0.3	1338 ±185	0.8	**t = -3.37** **p< 0.0001**	820 ±50	0.4
Number of collectors *per* species	4.6 ±0.3	2	5.3 ±0.9	2	t = -0.65 p = 0.5	4.7 ±0.2	2
Number of sampling dates for each species	6.7 ±0.4	3	6.7 ±0.7	3	t = 0.11 p = 0.9	7.7 ±0. 4	4
Number of specimens *per* species	37.5 ±5.5	7	26.4 ±4.5	7	t = 1.58 p = 0.1	35.9 ± 4	7
Number of sampling dates a species’ collector was in the field	105 ±4	44	89 ±9	32	t = 1.58 p = 0.1	103 ±3.8	27
Number of points sampled by a species’ collector	62.8 ±2.5	31	56.3 ±5.2	31	t = 1.12 p = 0.2	61.9 ±2.2	31
Area surveyed by a species’ collector (km^2^)	3,8 ±120	3	4,6 ±382	3,6	t = -1.85 p = 0.066	4,0 ±117	3

### c) Relationship between distribution range and sampling effort

The relationship between area and sampling effort was very similar in New Caledonian endemic and non-endemic species. In all cases, the correlation coefficient *r* between the area and sampling effort was low (Table 3). Nevertheless, there was a statistical difference between the sampling variables dealing with actual sampling and those dealing with potential sampling. On the one hand, two of the three variables describing the way the species were sampled (the number of collectors and the number of sampling dates *per* species) were significantly correlated with area. The number of collected specimens was the exception. On the other hand, the variables associated with the possibility of being collected were not found to be correlated with area (Table 3).

**Table 3:** Pearson’s correlation coefficient between area and different sampling effort variables. Significant correlations are indicated in bold.

	Endemic	Non-endemic	Total
*Variables derived from the information of collection of each species*			
Number of collectors	**r = 0.171** **p≤ 0.0001**	r = 0.140 p = 0.077	**r = 0.162** **p≤ 0.0001**
Number of sampling dates	**r = 0.211** **p≤ 0.0001**	**r = 0.261** **p = 0.0007**	**r = 0.220** **p≤ 0.0001**
Number of specimen *per* species	r = 0.017 p = 0.59	r = 0.077 p = 0.32	r = 0.020 p = 0.49
*Variables inferring to the possibility of being collected*			
Number of dates the species’ collectors were in the field	r = 0.004 p = 0.8	r = 0.403 p = 0.6	r = 0.013 p = 0.6
Number of points sampled by a species’ collectors	r = -0.011 p = 0.7	r = 0.022 p = 0.8	r = -0.006 p = 0.8
Mean of area prospected by a species’ collectors (km^2^)	r = -0.019 p = 0.56	r = 0.024 p = 0.76	r = 0.021 p = 0.48

### d) Choosing a variable to characterize the sampling effort in the dataset

The two variables that were significantly correlated with distribution range (the number of sampling dates and the number of collectors) were only slightly correlated when considering all species together (*r* = 0.058, p = 0.05), and not significantly correlated when considering endemics and non-endemics separately (*r* = 0.031, p = 0.32 for endemics) and (*r* = 0.131, p = 0.09 for non-endemics). This confirms, as expected, their independence.

**Fig 2:**
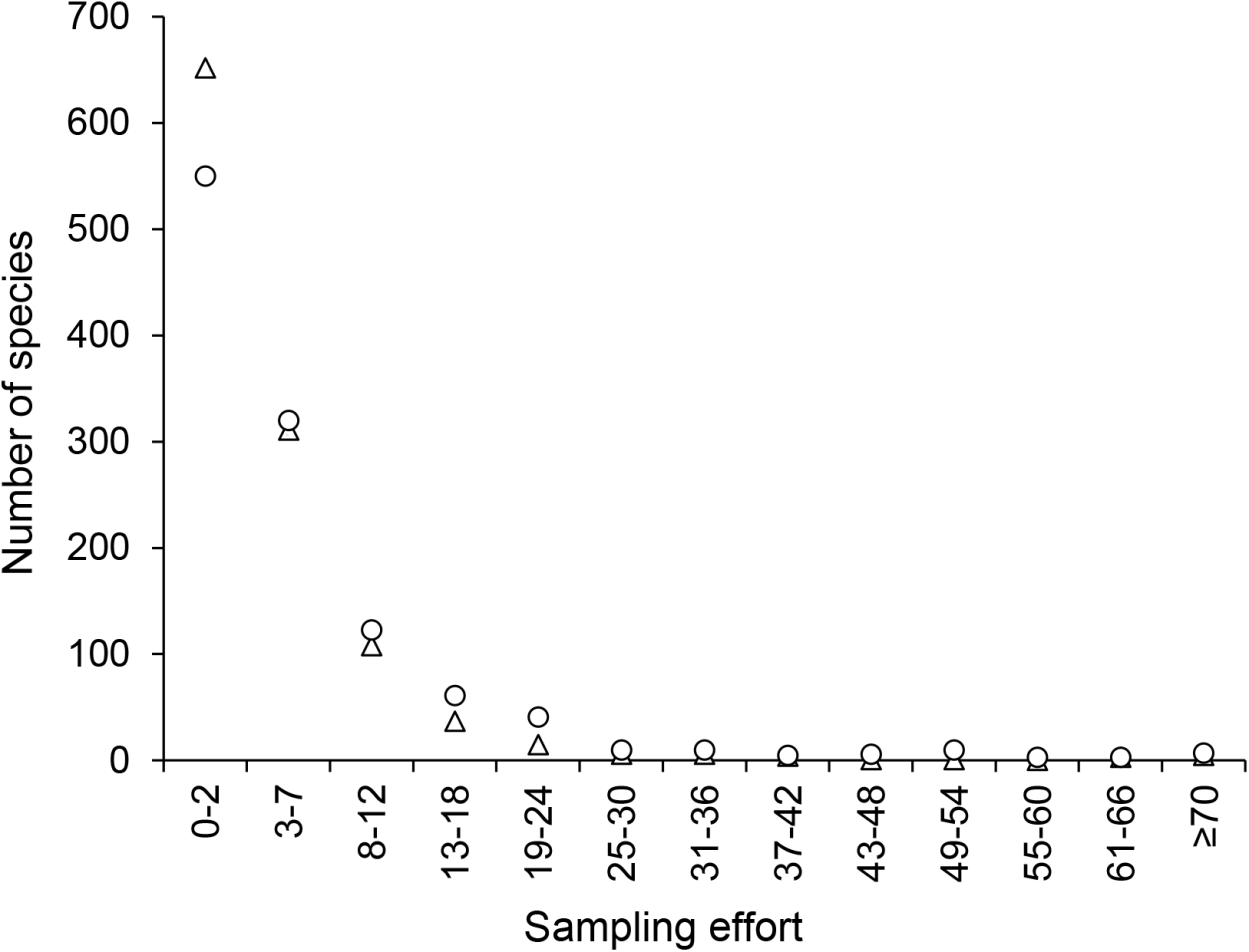
Number of species collected in different classes of sampling effort: triangles = number of collectors; circles = number of sampling dates (n = 1,149).

Nevertheless, when considering the frequency of species in different classes of sampling effort, a strong correlation between the number of sampling dates and the number of collectors was observed (*r* = 0.964, *r* = 0.897, *r* = 0.966, for endemic, non-endemic, and all species, respectively, with p< 0.00001 in all cases). Species sampled in very few dates or by very few collectors were the most frequent, followed by a marked decrease in frequency in subsequent classes (Fig 2).

**Fig 3:**
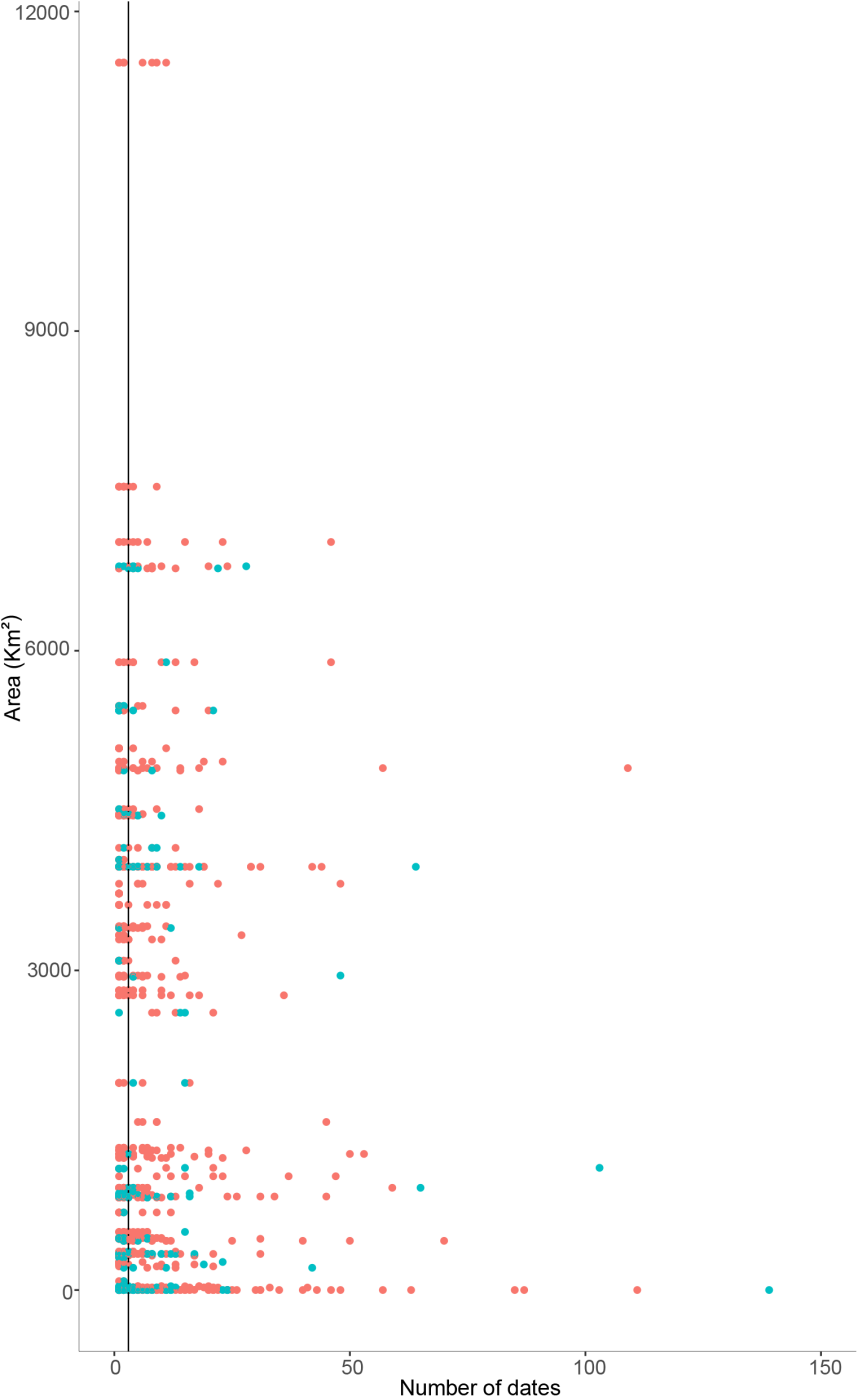
Distribution range in relation to the number of times the species was sampled for endemics (red) and non-endemics in New Caledonia (blue). The black line indicates the median sampling date (n = 3), used as a cut-off point between species that are considered to be correctly sampled and those that are considered data deficient.

Based on the strong correlation between these two variables, and considering that dates had stronger correlation coefficients with area, we used the number of sampling dates as a measure of the sampling effort in subsequent analyses. In addition, we used the median number of sampling dates from the entire dataset (= 3) as a cut-off point for establishing the adequacy of sampling for the inference of distribution range. That is, a species was considered adequately sampled if it was collected on at least 3 different sampling dates (Fig 3).

The relationship between distribution range and the number of sampling dates roughly described a triangular shape. This is due to the fact that a) the number of species collected with very low sampling effort was very high in all classes of distribution range; b) the number of species with a narrow distribution range was very high in all classes of sampling effort; and c) the number of species between these two extremes was much smaller (Fig 3).

### e) Distribution range of adequately sampled species

**Fig 4:**
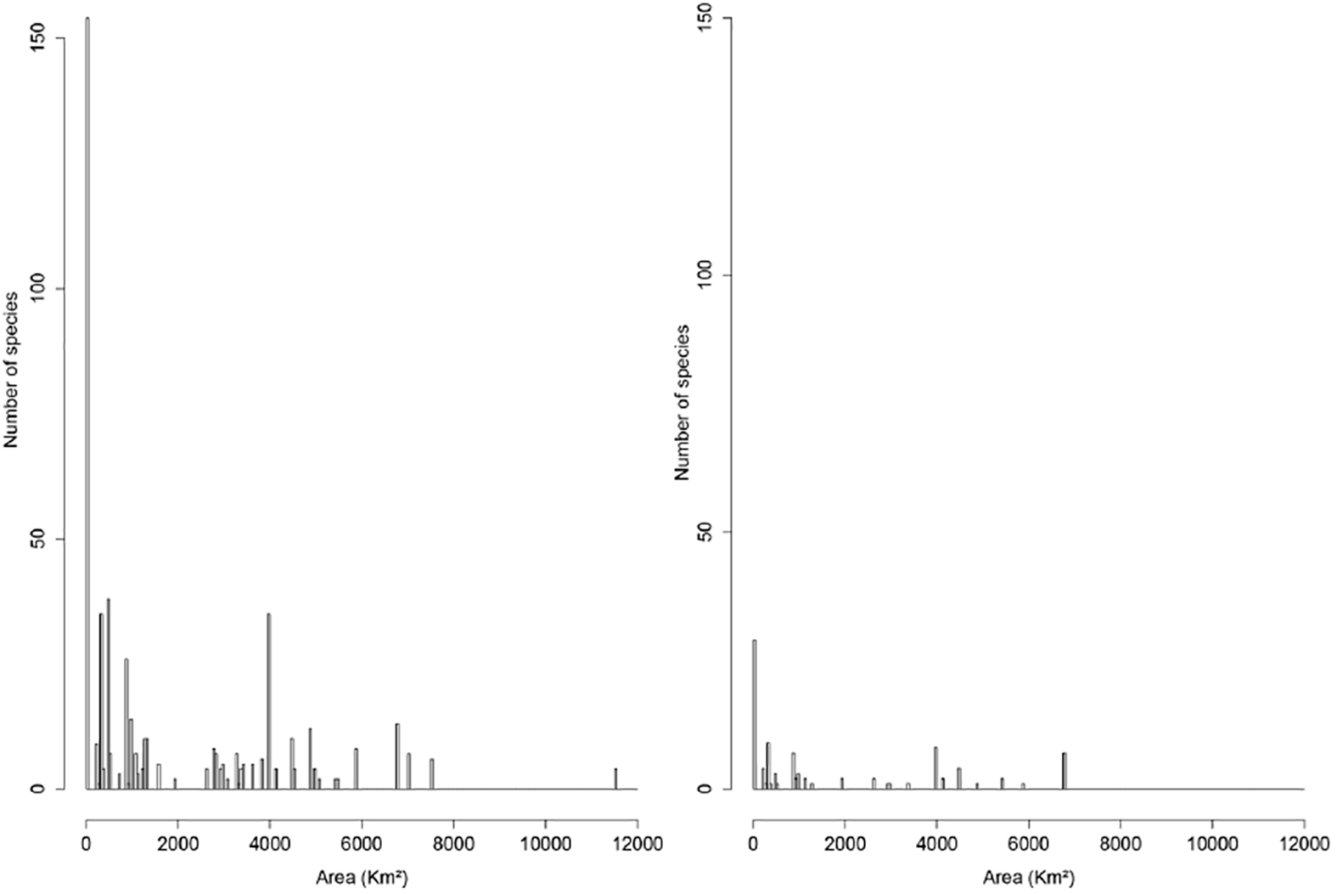
Number of endemic (A) and non-endemic (B) species sampled on at least 3 sampling dates in different classes of distribution range.

51% (502) of endemic and 57% (95) of non-endemic species were sampled a number of times equal to or higher than the median (i.e. ≥ 3 sampling dates) (Table 4). Despite the difference in total number, species with small distribution ranges were the most frequent in the two datasets (Fig 4A and 4B). 116 endemic species (12%) had distribution area ≤ 5.2 km^2^, and 38 (3.9%) occurred in areas between 23–100 km^2^. The percentage of non-endemics with a small distribution range in New Caledonia was much higher (22%–36 species—had a distribution area ≤ 5.2 km^2^, and 8% (13 species) had a distribution range between 23–100 km^2^) (S1 Table and S2 Table).

**Table 4:** The taxonomic orders analyzed in this study and their respective number of endemic and non-endemic species. Percentage values not in parentheses are for the total of each order. Percentage values in parentheses are based on the total number of species of the corresponding subset considered (total or sampled at least 3 times).

	Total			Sampled at least 3 times		
Order	Total	Endemic	Non- Endemic	Total	Endemic	Non-Endemic
Acari	60	52–86% (5%)	8–13% (5%)	38	35–67% (7%)	3–37% (3%)
Araneae	36	36–100% (3%)	0	7	7–19% (1%)	0
Myriapoda	22	18–82% (2%)	4–18% (2%)	11	8–44% (1%)	3–75% (3%)
Mollusca	27	27–100% (3%)	0	18	18–67% (3%)	0
Crustacea	37	17–43% (2%)	21–56% (12%)	16	7–41% (1%)	9–42% (9%)
Coleoptera	141	132–93% (14%)	9–6% (5%)	72	67–50% (13%)	5–55% (5%)
Collembola	87	57–65% (6%)	29–34% (17%)	36	20–35% (4%)	16–55% (17%)
Dictyoptera	34	34–100% (3%)	0	16	16–47% (3%)	0
Phasmatodea	14	11–78% (1%)	3–21% (2%)	8	5–45% (1%)	3–100% (3%)
Diptera	419	353–84% (36%)	67–16% (40%)	249	210–50% (42%)	39–58% (41%)
Hemiptera	79	65–82% (7%)	13–18% (8%)	38	29–45% (6%)	9–69% (9%)
Homoptera	14	14–100% (1%)	0	8	8–57% (1%)	0
Lepidoptera	34	34–100% (3%)	0	2	2–6% (0.3%)	0
Orthroptera	45	45–100% (5%)	0	27	27–60% (5%)	0
Neuroptera	1	1–100% (0.1%)	0	1	1–100% (0.1%)	0
Trichoptera	5	5–100% (0.5%)	0	3	3–60% (0.5%)	0
Opiliones	13	13–100% (1%)	0	5	5–38% (1%)	0
Tricladida	1	1–100% (0.1%)	0	1	1–100% (0.1%)	0
Teleostei	9	1–11% (0.1%)	8–88% (5%)	5	0	5–62% (5%)
Squamata	71	66–93% (7%)	5–7% (3%)	36	33–50% (6%)	3–60% (3%)
**Total**	**1149**	**982 (85%)**	**167 (15%)**	**597**	**502 (51%)**	**95 (57%)**

It is to be noted that although the distribution range of the set of species examined varied widely (see Table 2), there were major gaps in this continuum, with some classes of area without any species (see Fig 3). Hence, these gaps helped us define cut-off points for the different distribution range classes. This is how we arrived at the value of ≤5.2km^2^ for micro-endemic species. Similarly, the next group of species had distribution ranges varying from 23 km^2^ to 89 km^2^, which we synthetized as 23–100 km^2^.

### f) Contribution of organisms from different orders

Among the 20 orders represented in this dataset, Diptera, with 419 species, was by far the most diverse, followed by Coleoptera, Collembola and Squamata. All orders had at least one, often several, species endemic to New Caledonia, whereas the non-endemic species included here belonged to only 10 different orders. Diptera was also the richest in terms of number of species endemic to New Caledonia, followed by Coleoptera, Squamata and Hemiptera. Non-endemics were present in relatively high numbers in Diptera, Collembola and Crustacea, whereas they were poorly represented or absent in the other orders. The proportion of species endemic to New Caledonia was very high for all orders, except for Crustacea (43%) and Collembola (65%).

Despite the marked difference in the total number of endemic and non-endemic species, the proportion of species sampled on 3 or more sampling dates was quite high for several orders, for both endemic (51% of the total) and non-endemic species (57% of the total) (Table 4).

**Fig 5:**
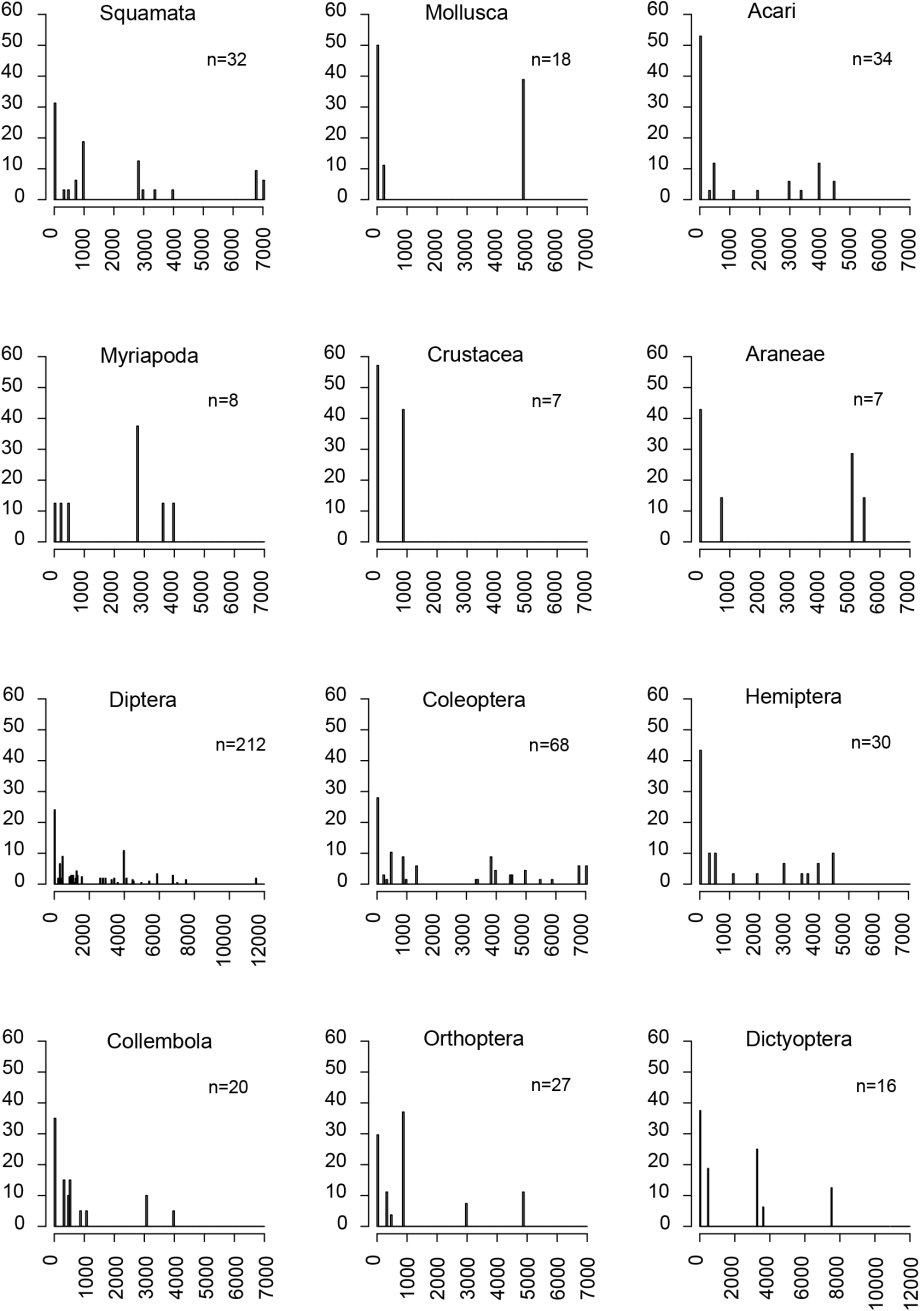
Frequency (%) of species endemic to New Caledonia in different classes of distribution range for 12 different orders.

The frequency of species from the most abundant orders across different classes of distribution range is presented in Fig 5. We found that high frequency in very small distribution ranges was common to almost all orders (except for Myriapoda and Orthoptera). In addition, only Diptera had species with the maximum distribution range estimated in this dataset (11,519km^2^). Squamata and Coleoptera also had species with large distribution ranges (up to 7,020km^2^). In the other orders, species distribution range never exceeded 5,500km^2^.

### g) Conservation status in the IUCN red list

Investigation of the IUCN red list shows that the number of species from our database that have been already assessed is very low (only 76 out of the 1,149) and that only a few groups of organisms are represented (Crustacea, Teleostei and Squamata). In addition, only Squamata species have an assessment with detailed information. Crustacea and Teleostei were only classified as LC (least concern) or DD (data deficient) (Table 5). Concerning the micro-endemic species only 15 are included in the IUCN red list, and only 12 occur in protected areas (S1 Table and S2 Table).

**Table 5:** The number of species within different categories of extinction risk in the IUCN red list grouped according to sampling adequacy (as established in this study) and distribution range estimates. Only three orders from the present dataset have been assessed by the IUCN. DD = data deficient; LC = least concern; NT = near threatened; VU = vulnerable; EN = endangered; CR = critically endangered.

	DD	LC	NT	VU	EN	CR	Listed IUCN	Total this study
Sampled in 1 or 2 dates								
Crustaceae	11	-	-	-	-	-	11	21
Teleostei	1	2	-	-	-	-	3	4
Squamata	6	1	6	3	4	4	24	36
	Sampled in ≥3 dates, ≤5.2 km^2^							
Crustaceae	-	-	-	-	-	-	-	1
Teleostei	2	-	-	-	-	-	2	2
Squamata	2	2	1	-	1	2	8	10
Sampled in ≥3 dates, ≥23 ≤100 km^2^								
Crustaceae	1	6	-	-	-	-	7	8
Teleostei	-	-	-	-	-	-	-	-
Squamata	-	-	-	-	-	-	-	1
Sampled in ≥3 dates, >100km^2^								
Crustaceae	-	4	-	-	-	-	4	6
Teleostei	-	2	-	-	-	-	2	3
Squamata	-	2	3	3	3	4	15	24

## Discussion

Although micro-endemicity has frequently been reported in researches for different groups of the New Caledonian fauna [10, 14, 16, 23, 26–27, 31], the present study is the first to be specifically designed to quantify its distribution range, estimate its frequency, and determine the extent to which these findings are common to several taxonomic groups. It is also the first time that the distribution range of this fauna is analyzed taking sampling effort into account.

The dataset we used was certainly incomplete in terms of the species covered. However, it had the advantage of including a large number of orders, comprising organisms with different life habits, sizes and natural histories. In addition, it included a high number of species that were new to science. Our dataset was compiled from species descriptions that were done within an explicit taxonomic framework, which means that scientific names and specimen occurrence data were based on the work of experts. This is undoubtedly preferable to using second hand faunistic compilations or natural history collection databases that often include uncertain taxonomic treatments and doubtful identifications.

### Sampling effort variables and their implications on estimated distribution ranges

Among the sampling effort variables examined, only the number of times the species was sampled and the number of collectors were correlated with area. This highlights the importance of diversity in the way of sampling in order to increase the probability finding a wider distribution range for a given species. This result is in accordance with the topography and distribution of remnants of different forests in New Caledonia, which are marked by important discontinuities. The relief formed by the central chain, which is divided into a series of mountains with steep slopes, certainly constrains the mobility of fieldworkers. This makes it difficult to increase the area surveyed without moving from one slope to the other, or from one mountain to the next, which requires long walks or driving to access sampling sites from another flank. Thus, researchers often focus on nearby spots on a same day, and start surveying more distant sites at another time. The fact that some forest remnants are distributed across several scattered patches also contributes to that surveys are spread out over time. As expected from previous studies on the effect of sampling conditions, accessibility constraints have an impact on specific richness estimates [45, 46].

The fact that the number of specimens for a given species was not correlated with area was not a surprise, as the number of specimens is often a choice of the collectors. However, the low figures observed here suggest that in general densities are low, which is in accordance with our (PG, RP) field observations and the repeated remarks in the literature [16]. Nevertheless, more studies on population ecology are required to confirm the extent to which low population densities are prevalent across taxa in New Caledonia.

Unlike the actual sampling effort variables, we found a lack of relationship between the variables that relate to the possibility of being collected and area, which was unexpected. Intuitively, one would expect that the more the collectors sampled the territory, the higher the chances of finding a species in the entire range of its occurrence. Therefore, our interpretation for the lack of relationship between area and these variables (i.e. number of dates, number of points, area covered by all the collectors that sampled the species) is that, for many species, the estimated distribution range might be very close to reality and not a mere sampling artifact.

Another important question in this analysis was the definition of a cut-off point above which the sampling would be considered adequate. We chose to use the median sampling effort. Although arbitrary, this allows for comparisons between different datasets.

### Extent of micro-endemicity

Our results show that the rate of micro-endemicity is indeed extremely high. If we had based our inferences on the entire dataset, 22% of the species endemic to New Caledonia could be considered to be very narrow endemics (with a distribution range no larger than 5.2km^2^). This figure is quite similar to the estimates of Wulff *et al*. [32], who showed that 21.7% of plants in New Caledonia are found in at least three different localities and over less than 10km^2^ [32].

By considering the impact of sampling effort on distribution range estimates, we refined these estimates, which led to considerations about the nature of the distribution of this fauna. The set of species that were sampled well enough to allow a sound inference of their distribution range was much smaller than the total number of species assessed. This brings the rates of confirmed micro-endemism down to 12%, (116 species with areas ≤ 5.2 km^2^). In addition, 3.9% of these well-studied species also occur in small areas (ranging from 23–100 km^2^).

Our study suggests that micro-endemism represents a general characteristic of the New Caledonian fauna, which is consistent with what has been found for the New Caledonian flora [32]. The extreme scale of this micro-endemism is remarkable as it probably involves extremely small populations, given the prevalence of small areas and low densities. Previous case studies of various groups showed that these species are well-differentiated from a morphological, molecular or behavioral point of view [22–25]. Therefore, micro-endemism arose from normal evolutionary differentiation equivalent to other highly diverse but less micro-endemic faunas.

### What about non-endemic species?

A surprising result in this study concerns species that are not endemic to New Caledonia. Even if on average they had larger distribution areas (Table 2), an important fraction had very restricted ranges (22% in areas ≤ 5.2 km^2^, and 8% in areas 23–100 km^2^). Several hypotheses might explain these restricted distributions. The most likely is a) that their niches are clumped together on the island, or 2) that some of them are occasional species, which are typically found in low abundance with different habitat requirements [47]. Considering the present dataset, some of these species would be classified as common-rare-rare by Rabinowitz [1], meaning that, although they are found across distant locations, their total distribution is due to several, but very localized populations. More data on their distribution range could clarify this point. However, it highlights the difficulty of estimating distribution range and, consequently, of defining categories of threat in species with this pattern of distribution (see [43] for a discussion of this topic).

As a perspective for future research, this result should help in the interpretation of the role of environmental factors and in understanding the origin of micro-endemism in local species. The restricted distribution areas of non-endemic species could mean that distribution is primarily controlled by environmental parameters and their small-scale variation in a patchy landscape.

### Distribution and conservation status

As already stated by Wulff *et al*. [32] concerning the New Caledonian flora, our results indicate that the IUCN red list needs an urgent re-assessment concerning the endemic fauna of New Caledonia. Our results provide estimates of the distribution range in a wide range of species, which will facilitate the re-assessment of the status of species from at least 12 different orders (S1 Table and S2 Table). Considering the ongoing environmental pressure and destruction of several habitats in New Caledonia [10, 19–23], the fact that all species with a distribution range <10 km^2^ occur in a single locality, and that population sizes are frequently very small, and that very few of these species occur in areas legally protected, a great number of these micro-endemic species are very likely to be Critically Endangered.

In conclusion, our study shows that the New Caledonian biota is composed of very narrowly distributed species. Based on existing information, 12% of the species were characterized as micro-endemic, with known distribution ranges no greater than 5.2 km^2^. Nearly 4% of species were shown to occur in areas smaller than 100 km^2^. This result is consistent with a previous assessment for the flora. By taking the sampling effort variables into account, we were able to show that these distribution patterns are not a spurious effect of undersampling but are the hallmark of rarity in a constraining landscape. This study sets up the foundation for a global study of micro-endemism, which should now focus on the environmental parameters that possibly determined its evolution. This would represent an invaluable source for future phylogeographic studies and for establishing policies of land management and biodiversity conservation.

## Supporting information

S1 Table (DOCX)

S2 Table (DOCX)
