# Supplementary material for: Outstanding micro-endemism in New Caledonia: More than one out of ten animal species have a very restricted distribution range
Source: PLoS One. 2017 Jul 20;12(7):e0181437. doi: 10.1371/journal.pone.0181437 (PMC5519078; doi:10.1371/journal.pone.0181437)
Supplement: S1 Table — (DOCX) [file pone.0181437.s001.docx]

**Table S1. List of 116 species with distribution area ≤5.2km², with respective taxonomic hierarchy, conservation status in the IUCN red list (IUCN) and their occurrence in protected areas (PA).**

| **Order** | **Family** | **Species** | **IUCN** | **PA** |
| --- | --- | --- | --- | --- |
| Coleoptera | Curculionidae | *Achorius parvioculis* |  |  |
| Orthoptera | Gryllidae | *Adenopterus amiensis* |  |  |
| Myriapoda | Pauropodidae | *Allopauropus macropygus* |  |  |
| Myriapoda | Pauropodidae | *Allopauropus tillierae* |  |  |
| Dictyoptera | Blattidae | *Angustonicus boucheti* |  |  |
| Squamata | Diplodactylidae | *Oedodera aff. marmorata* | LC |  |
| Hemiptera | Meenoplidae | *Anorhinosia anomala* |  |  |
| Squamata | Scincidae | *Sigaloseps conditus* | LC |  |
| Coleoptera | Belidae | *Aralius dispar* |  |  |
| Collembola | Onychiuridae | *Boudinotia prima* |  |  |
| Coleoptera | Staphylinidae | *Brachida stilifera* |  |  |
| Orthoptera | Gryllidae | *Bullita fusca* |  |  |
| Dictyoptera | Blattidae | *Rothisilpha panie* |  |  |
| Crustacea | Armadillidae | *Caledonillo bruneum* |  |  |
| Crustacea | Armadillidae | *Caledonillo tillierorum* |  |  |
| Hemiptera | Meenoplidae | *Caledonisia bolanus* |  |  |
| Squamata | Diplodactylidae | *Eurydactylodes vieillardi* | NT |  |
| Hemiptera | Meenoplidae | *Caledonisia monsmollis* |  |  |
| Hemiptera | Meenoplidae | *Caledonisia theophane* |  |  |
| Diptera | Drosophilidae | *Scaptodrosophila loxostyla* |  |  |
| Orthoptera | Gryllidae | *Koghiella bouleti* |  |  |
| Phasmatodea | Phasmatidae | *Gigantophasma bicolor* |  |  |
| Orthoptera | Phalangopsidae | *Caltathra chopardi* |  |  |
| Orthoptera | Phalangopsidae | *Caltathra spp.* |  |  |
| Orthoptera | Phalangopsidae | *Caltathra areto* |  |  |
| Myriapoda | Pauropodidae | *Hemipauropus melanesicus* |  | Rivière Bleue Provincial Park |
| Hemiptera | Tingidae | *Cephalidiosus sineorœ* |  |  |
| Hemiptera | Tingidae | *Cephalidiosus thy* |  |  |
| Diptera | Tabanidae | *Chasmia bozennae* |  |  |
| Homoptera | Eriococcidae | *Chazeauana gahniae* |  |  |
| Diptera | Tipulidae | *Styringomyia bidentata* |  |  |
| Diptera | Dolichopodidae | *Corindia flaviscuta* |  |  |
| Hemiptera | Tingidae | *Corinthus plumensis* |  |  |
| Collembola | Keroplatidae | *Pyrtulina dubia* |  |  |
| Diptera | Tabanidae | *Cydistomyia brachypalpus* |  |  |
| Diptera | Tabanidae | *Cydistomyia massali* |  |  |
| Coleoptera | Staphylinidae | *Cypha lancea* |  |  |
| Diptera | Drosophilidae | *Scaptodrosophila sp. 10* |  |  |
| Hemiptera | Meenoplidae | *Distantiana stylirecta* |  |  |
| Coleoptera | Curculionidae | *Dracophyllius marginicollis* |  |  |
| Diptera | Neanuridae | *Ectonura koumac* |  |  |
| Diptera | Drosophilidae | *Scaptodrosophila sp. 12* |  |  |
| Collembola | Neanuridae | *Ectonura paralata* |  |  |
| Collembola | Neanuridae | *Ectonura proxima* |  |  |
| Collembola | Neanuridae | *Ectonura pulchra* |  | Rivière Bleue Provincial Park |
| Collembola | Neanuridae | *Elytroteinus geophilus* |  |  |
| Diptera | Empididae | *Empis nondouensis* |  |  |
| Diptera | Tachinidae | *Senostoma flavipes* |  |  |
| Araneae | Barychelidae | *Encyocrypta berlandi* |  |  |
| Diptera | Barychelidae | *Encyocrypta cagou* |  |  |
| Diptera | Barychelidae | *Encyocrypta colemani* |  |  |
| Opiliones | Troglosironidae | *Troglosiro brevifossa* |  | Cap N’Dua Natural Reserve |
| Opiliones | Troglosironidae | *Troglosiro oscitatio* |  |  |
| Opiliones | Troglosironidae | *Troglosiro raveni* |  |  |
| Diptera | Barychelidae | *Encyocrypta koghi* |  |  |
| Hemiptera | Meenoplidae | *Suvanisia caudafurcata* |  |  |
| Trichoptera | Hydrobiosidae | *Xanthochorema christinae* |  |  |
| Diptera | Barychelidae | *Encyocrypta kritscheri* |  |  |
| Squamata | Scincidae | *Phoboscincus bocourti* |  |  |
| Coleoptera | Staphylinidae | *Lomaglossina domina* |  | Rivière Bleue Provincial Park |
| Diptera | Dolichopodidae | *Parentia webbi* |  | Rivière Bleue Provincial Park |
| Crustacea | Atyidae | *Paratya typa* |  |  |
| Homoptera | Tibicinidae | *Ueana desserti* |  |  |
| Diptera | Keroplatidae | *Neoplatyura lyraefera* |  |  |
| Diptera | Tipulidae | *Toxorhina juvenca* |  |  |
| Squamata | Scincidae | *Nannoscincus exos* | CR |  |
| Coleoptera | Phalangopsidae | *Tremellia aptera* |  |  |
| Coleoptera | Dytiscidae | *Exocelina nehoue* |  |  |
| Hemiptera | Meenoplidae | *Fennahsia cafana* |  |  |
| Hemiptera | Meenoplidae | *Fennahsia matuta* |  |  |
| Hemiptera | Meenoplidae | *Fennahsia perioflata* |  |  |
| Collembola | Isotomidae | *Folsomides insularis* |  |  |
| Diptera | Mycetophilidae | *Caledonileia pusilla* |  |  |
| Diptera | Bombyliidae | *Geron aaptis* |  |  |
| Hemiptera | Meenoplidae | *Glyptodonisia tomyris* |  |  |
| Diptera | Dolichopodidae | *Lapita cudo* |  |  |
| Coleoptera | Staphylinidae | *Sternotropa multiplex* |  |  |
| Diptera | Dolichopodidae | *Lapita expirata* |  |  |
| Diptera | Tipulidae | *Leptotarsus glabristylus* |  |  |
| Diptera | Dolichopodidae | *Lapita laniensis* |  |  |
| Diptera | Dolichopodidae | *Lapita schlingeri* |  |  |
| Diptera | Ditomyiidae | *Nervijuncta vicina* |  |  |
| Diptera | Dolichopodidae | *Lapita semita* |  |  |
| Diptera | Mycetophilidae | *Leia delobeli* |  |  |
| Teleostei | Gobiidae | *Stiphodon mele* | DD |  |
| Teleostei | Gobiidae | *Stiphodon semoni* | DD |  |
| Diptera | Dolichopodidae | *Pouebo symmetricauda* |  |  |
| Squamata | Scincidae | *Simiscincus aurantiacus* |  |  |
| Coleoptera | Apionidae | *Megatracheloides millei* |  |  |
| Diptera | Tipulidae | *Limonia ovalistigma* |  |  |
| Trichoptera | Hydrobiosidae | *Xanthochorema johnwardi* |  |  |
| Squamata | Diplodactylidae | *Dierogekko baaba* | CR |  |
| Squamata | Diplodactylidae | *Bavayia cyclura clade* | EN |  |
| Diptera | Dolichopodidae | *Corindia amieuensis* |  |  |
| Diptera | Tabanidae | *Dasybasis danielae* |  |  |
| Diptera | Tabanidae | *Dasybasis tillierorum* |  |  |
| Diptera | Ceratopogonidae | *Dasyhelea neocaledoniensis* |  |  |
| Diptera | Keroplatidae | *Dimorphelia stirpicola* |  |  |
| Diptera | Keroplatidae | *Dimorphelia tergata* |  |  |
| Collembola | Onychiuridae | *Dinaphorura chazeaui* |  |  |
| Collembola | Onychiuridae | *Dinaphorura matileorum* |  |  |
| Collembola | Neanuridae | *Ectonura minima* |  |  |
| Diptera | Barychelidae | *Encyocrypta panie* |  |  |
| Diptera | Ceratopogonidae | *Forcipomyia pectinunguis* |  |  |
| Diptera | Dolichopodidae | *Lapita ternata* |  |  |
| Diptera | Drosophilidae | *Mycodrosophila legrandi* |  |  |
| Hemiptera | Tingidae | *Nobarnus typicus* |  |  |
| Coleoptera | Scirtidae | *Scirtes ebenus* |  |  |
| Lepidoptera | Micropterigidae | *Sabatinca delobeli* |  |  |
| Coleoptera | Curculionidae | *Anomalodermus proclivis* |  |  |
| Diptera | Mycetophilidae | *Anomalomyia sp.* |  |  |
| Diptera | Dolichopodidae | *Antyx pallidiventris* |  |  |
| Orthoptera | Gryllidae | *Bullita transversa* |  |  |
| Orthoptera | Gryllidae | *Bullita unicolor* |  |  |
| Dictyoptera | Blattidae | *Rothisilpha panie* |  |  |
| Hemiptera | Meenoplidae | *Caledonisia crypta* |  |  |
