# Supplementary material for: Outstanding micro-endemism in New Caledonia: More than one out of ten animal species have a very restricted distribution range
Source: PLoS One. 2017 Jul 20;12(7):e0181437. doi: 10.1371/journal.pone.0181437 (PMC5519078; doi:10.1371/journal.pone.0181437)
Supplement: S2 Table — (DOCX) [file pone.0181437.s002.docx]

**Table S2. List of 39 species with distribution area between 10 and 100km². with respective taxonomic hierarchy, conservation status in the IUCN red list (IUCN) and their occurrence in protected areas (PA).**

| **Order** | **Family** | **Species** | **IUCN** | **PA** |
| --- | --- | --- | --- | --- |
| Mollusca | Helicinidae | *Sturanya dautzenbergi* |  |  |
| Mollusca | Helicinidae | *Sturanya gassiesiana* |  |  |
| Mollusca | Helicinidae | *Sturanya koumacensis* |  |  |
| Mollusca | Helicinidae | *Sturanya laeta* |  |  |
| Mollusca | Helicinidae | *Sturanya littoralis* |  |  |
| Mollusca | Helicinidae | *Sturanya macgillivrayi* |  |  |
| Araneae | Dipluridae | *Caledothele tristata* |  |  |
| Orthoptera | Gryllidae | *Calscirtus amoa* |  | Vallée de la Thy Natural Reserve |
| Orthoptera | Phalangopsidae | *Caltathra areto* |  |  |
| Orthoptera | Phalangopsidae | *Caltathra doensis* |  |  |
| Orthoptera | Phalangopsidae | *Caltathra steinmanni* |  | Vallée de la Thy Natural Reserve |
| Phasmatodea | Phasmatidae | *Canachus alligator* |  | Rivière Bleue Provincial Park |
| Phasmatodea | Phasmatidae | *Canachus crocodilus* |  |  |
| Phasmatodea | Phasmatidae | *Canachus harpya* |  |  |
| Crustacea | Atyidae | *Caridina gracilirostris* | LC |  |
| Crustacea | Atyidae | *Caridina imitatrix* | LC | Rivière Bleue Provincial Park |
| Crustacea | Atyidae | *Caridina leucosticta* | LC |  |
| Crustacea | Atyidae | *Caridina longirostris* | LC |  |
| Crustacea | Atyidae | *Caridina nilotica* | LC |  |
| Crustacea | Atyidae | *Caridina novaecaledoniae* | LC |  |
| Crustacea | Atyidae | *Caridina vitiensis* | DD |  |
| Collembola | Brachystomellidae | *Cassagnella sp.* |  |  |
| Squamata | Scincidae | *Celatiscincus euryotis* |  |  |
| Hemiptera | Tingidae | *Cephalidiosus sineorœ* |  |  |
| Hemiptera | Tingidae | *Cephalidiosus spinosus* |  |  |
| Diptera | Tephritidae | *Ceratitella schlingeri* |  |  |
| Collembola | Neanuridae | *Ceratrimeria lydiae* |  |  |
| Diptera | Tabanidae | *Chasmia cohici* |  |  |
| Diptera | Tabanidae | *Chasmia leszeki* |  |  |
| Homoptera | Eriococcidae | *Chazeauana gahniae* |  |  |
| Diptera | Mycetophilidae | *Leia delobeli* |  |  |
| Collembola | Entomobryidae | *Lepidocyrtus sp.* |  |  |
| Diptera | Tephritidae | *Dirioxa pornia* |  |  |
| Hemiptera | Meenoplidae | *Distantiana stylirecta* |  |  |
| Coleoptera | Curculionidae | *Dracophyllius dracophylli* |  |  |
| Diptera | Drosophilidae | *Drosophila kanaka* |  | Vallée de la Thy Natural Reserve |
| Diptera | Drosophilidae | *Drosophila levii* |  | Vallée de la Thy Natural Reserve |
| Diptera | Drosophilidae | *Drosophila ochrogaster* |  |  |
| Diptera | Drosophilidae | *Drosophila serrata* |  | Vallée de la Thy Natural Reserve |
